# Role of Runx2 in Calcific Aortic Valve Disease in Mouse Models

**DOI:** 10.3389/fcvm.2021.687210

**Published:** 2021-10-29

**Authors:** Subramanian Dharmarajan, Mei Y. Speer, Kate Pierce, Jake Lally, Elizabeth M. Leaf, Mu-En Lin, Marta Scatena, Cecilia M. Giachelli

**Affiliations:** Department of Bioengineering, University of Washington, Seattle, WA, United States

**Keywords:** RUNX2, CAVD, calcification, aortic valve stenosis, valve interstitial cells, osteochondrogenic differentiation

## Abstract

**Background:** Calcific aortic valve disease is common in the aging population and is characterized by the histological changes of the aortic valves including extracellular matrix remodeling, osteochondrogenic differentiation, and calcification. Combined, these changes lead to aortic sclerosis, aortic stenosis (AS), and eventually to heart failure. Runt-related transcription factor 2 (Runx2) is a transcription factor highly expressed in the calcified aortic valves. However, its definitive role in the progression of calcific aortic valve disease (CAVD) has not been determined. In this study, we utilized constitutive and transient conditional knockout mouse models to assess the molecular, histological, and functional changes in the aortic valve due to Runx2 depletion.

**Methods:** Lineage tracing studies were performed to determine the provenance of the cells giving rise to Runx2+ osteochondrogenic cells in the aortic valves of LDLr^−/−^ mice. Hyperlipidemic mice with a constitutive or temporal depletion of Runx2 in the activated valvular interstitial cells (aVICs) and sinus wall cells were further investigated. Following feeding with a diabetogenic diet, the mice were examined for changes in gene expression, blood flow dynamics, calcification, and histology.

**Results:** The aVICs and sinus wall cells gave rise to Runx2+ osteochondrogenic cells in diseased mouse aortic valves. The conditional depletion of Runx2 in the SM22α+ aVICs and sinus wall cells led to the decreased osteochondrogenic gene expression in diabetic LDLr^−/−^ mice. The transient conditional depletion of Runx2 in the aVICs and sinus wall cells of LDLr^−/−^ApoB^100^ CAVD mice early in disease led to a significant reduction in the aortic peak velocity, mean velocity, and mean gradient, suggesting the causal role of Runx2 on the progression of AS. Finally, the leaflet hinge and sinus wall calcification were significantly decreased in the aortic valve following the conditional and temporal Runx2 depletion, but no significant effect on the valve cusp calcification or thickness was observed.

**Conclusions:** In the aortic valve disease, Runx2 was expressed early and was required for the osteochondrogenic differentiation of the aVICs and sinus wall cells. The transient depletion of Runx2 in the aVICs and sinus wall cells in a mouse model of CAVD with a high prevalence of hemodynamic valve dysfunction led to an improved aortic valve function. Our studies also suggest that leaflet hinge and sinus wall calcification, even in the absence of significant leaflet cusp calcification, may be sufficient to cause significant valve dysfunctions in mice.

## Introduction

Calcific aortic valve disease, the progressive accumulation of fibrocalcific matrices and calcified nodules in the aortic valves that results in the narrowing of the aortic valve, termed aortic stenosis (AS), accounts for ~50% of cardiac valve diseases (1–3). In developed countries, calcific aortic valve disease (CAVD) is the third most common cardiovascular disease behind coronary artery disease and hypertension (2). While CAVD is often asymptomatic during the first several decades of life, notably, ~1/3 of our elderly have early valve disease as indicated by the echocardiographic or radiological evidence of aortic sclerosis (4–6). By the age of 65, ~2% of individuals develop symptomatic AS, characterized by severe valve calcification, impaired leaflet motion, and cardiac outflow, which, if untreated, results in left ventricular hypertrophy, angina pectoris, syncope, and heart failure (1–3, 5, 7). Indeed, patients with CAVD are exposed to a 50% increase in the risk of myocardial infarction and death from cardiovascular causes during the next 5 years (1, 5, 8). Patients with type II diabetes mellitus also have a heightened risk for CAVD (9–11).

Calcific aortic valve disease is characterized by the fibro-calcific changes in the aortic valve. The initial stage of the disease is characterized by the thickening and mild calcification of the valve leaflet, with little or no changes to the blood flow dynamics (aortic sclerosis). Over time, an increase in valve calcification leads to the loss of compliance, impaired leaflet motion, and obstruction to blood flow (AS) (12). Although initially considered a degenerative process resulting from the natural “wear-and-tear” of the valve leaflets, recent evidence supports CAVD as an actively regulated process, involving cells within the valve leaflets, annulus, and sinuses (2, 13, 14). During the early stages of aortic valve sclerosis, focal subendothelial lesions are found on the aortic side of leaflets as well as the sinus wall (15), and are characterized by endothelial disruption, inflammatory cell infiltration, and cholesterol accumulation (16, 17). In addition to sinus wall lesion cells, some valvular interstitial cells (VICs) become “activated” and express myofibroblast marker proteins, including smooth muscle α-actin (SMA) and SM22α (SM22) (16, 18, 19). Diseased valves also upregulate osteochondrogenic mediators and transcription factors, such as BMP2/4, Runx2, and Sox9 (20–22), and bone and cartilage matrix mineralization-related proteins, such as alkaline phosphatase (ALP), osteopontin (OPN), osteocalcin (OCN), and collagen types II and X (Col II and X) (21–25). As the disease advances, cartilaginous and bony metaplasia occur (20, 26), mimicking active bone formation and remodeling.

Runt-related transcription factor 2 is a master transcription factor critical for skeleton formation and the ectopic calcification of blood vessels (27). While runt-related transcription factor 2 (Runx2) is not normally expressed in the aortic valves and blood vessels, numerous studies have demonstrated the *de novo* expression of Runx2 associated with osteochondrogenic cell differentiation and the formation of cartilaginous and calcified bone-like lesions in aortic valve disease, diabetic calcifying lesions, and atherosclerotic plaques in humans and experimental animal models (21, 22, 24, 28–36). The interstitial cells isolated from the stenotic human aortic valve leaflets showed greater levels of BMP2 that, when added to the VICs derived from normal aortic valves, induced a marked increase in the Runx2 expression prior to the matrix calcification (37). Finally, Wirrig et al. observed activated VICs (aVICs), extracellular matrix (ECM) disorganization, and markers of mesenchymal and skeletal osteochondrogenic progenitor cells in both pediatric non-calcified and adult calcified aortic valves (22). Interestingly, the expression of Runx2 only occurred in adult calcified aortic valves (22), suggesting that Runx2 may be required for osteochondrogenic differentiation and calcification in the aortic valves. Furthermore, an RNA sequencing analysis comparing human aortic valve samples with and without AS revealed Runx2 as a potential driver of AS development (38).

In the present report, lineage-tracing studies and Runx2 constitutive and transient conditional knockout in hyperlipidemic mice were utilized to determine (1) the lineage of the cells giving rise to Runx2+ osteochondrogenic precursors in the aortic valve, (2) whether the Runx2 expression in the SMA+ valve cells is required for osteochondrogenic cell differentiation, and (3) whether the Runx2 expression in the SMA+ valve cells contributed to the progressive calcification and valve dysfunction in mice.

## Materials and Methods

### Animal Models

For the genetic lineage tracing studies, LDLr^−/−^:R26R^f/f^: SM22-Cre^+/o^ (LDLr^−/−^ β-Gal^SM22^) mice fed with a diabetogenic (T2DM) diet for 28 weeks were utilized as previously described (36). As previously shown, the T2DM diet (Bio-Serv, 1.25% cholesterol, 57.5% kcal fat, 27.4% kcal carbohydrate) induces obesity, hyperglycemia, hypertriglyceridemia, and hypercholesterolemia in LDLr^−/−^ mice (36, 39). Mice carrying conditional alleles that target the runt homology domain of the Runx2 gene (*Runx2 flox*) were generated as previously described (40). To generate mice with depletion of Runx2 in the SM22α+ aVICs and sinus wall cells, LDLr^−/−^:Runx2^ΔSM22^ and its control LDLr^−/−^:Runx2^f/f^, *Runx2 flox* mice were backcrossed with C57BL/6J wildtype mice three times prior to breeding with congenic C57BL/6J SM22α-Cre mice and LDLr^−/−^ mice. To generate mice with a transient depletion of Runx2 in the SMA+ aVICS and sinus wall cells, LDLr^−/−^ApoB^100^Runx2^f/f^: SMACreERT2^+/o^ (LDLr^−/−^ApoB^100^Runx2^ΔSMA^) and its control LDLr^−/−^ApoB^100^Runx2^f/f^ (LDLr^−/−^ApoB^100^Runx2^f/f^), *Runx2 flox* mice were backcrossed with C57BL/6J wildtype mice three times prior to breeding with congenic C57BL/6J SMA-CreERT2 mice and LDLr^−/−^ApoB^100^ mice. Male LDLr^−/−^ApoB^100^Runx2^ΔSMA^ and LDLr^−/−^ApoB^100^Runx2^f/f^ littermates, 10–12 weeks old (≥20 g), were randomly assigned to be fed with either a diabetogenic or a procalcific diet for 26 weeks (T2DM) to induce CAVD, or normal chow (NC) as dietary control (36, 39). The T2DM LDLr^−/−^ApoB^100^Runx2^ΔSMA^ and LDLr^−/−^ApoB^100^Runx2^f/f^ were injected with 50 mg/kg tamoxifen (20 μg/μl tamoxifen (Sigma T5648) dissolved in corn oil). Briefly, the tamoxifen was added to corn oil in sterile conditions and placed on a rotator at room temperature for 3 h prior to the injection to dissolve the tamoxifen completely. Genotyping was performed using the PCR of the DNA from the aortic valve to verify the Runx2 deletion. Primers used for genotyping are as described previously (40). The primers generated amplicons of 1,269 bp in the floxed conditional allele and 527 bp in Cre recombined allele. The body weight and blood glucose levels were recorded before the diet challenge, every 4 weeks following the diet switch, and at study termination. The fasted sera were collected before the diet switch and at termination. The mice were euthanized *via* an intraperitoneal injection of pentobarbital (150 mg/kg) followed by exsanguination through cardiac puncture. All animals were maintained in a specific pathogen-free environment and the genotypes were determined as described (36, 40). All protocols are in compliance with the National Institutes of Health (NIH) Guideline for the Care and Use of Laboratory Animals and have been approved by the Institutional Animal Care and Use Committee of the University of Washington.

### Echocardiography

Transthoracic echocardiography was performed in the isoflurane-anesthetized mice with a heart rate of ~400–500 beats/min using a high-resolution *in vivo* ultrasound imaging system for small animals equipped with a 40-MHz transducer (Vevo 2100™, VisualSonics Inc., Canada). The aortic valve function was assessed using the Pulse Wave Doppler-mode that measures the aortic valve peak velocity, mean velocity, peak gradient, and mean gradient. In brief, the Doppler flow velocity spectrum of the ascending aorta of each mouse was recorded at the anterior of the aortic lumen and the left ventricular outflow tract (LVOT). A minimum of three cardiac cycles at each location was traced to obtain an average for the aortic peak velocity and gradient using the Vevo 2100™ software. Images were taken from the upper right parasternal long-axis view and the angle of the transducer was maintained at 40–50 degrees. The mean gradient was calculated using the modified Bernoulli's equation [Δ*P* (*Mean gradient*) = 4 (*Velocity AoV*^2^-*VelocityLVOT T*^2^)]. The aortic valve area (AVA) was calculated using the velocity time integral (VTI) values in the continuity equation [$AVA=\frac{{LVOT\;}_{Diameter^{*}}VTI_{LVOT}}{VTI_{AoV}}$]. We also used B-mode and M-mode images of the parasternal long-axis view to measure the left ventricular dimensions and volumes, fractional shortening, and ejection fraction.

### Histochemical and Immunohistochemical Staining

The hearts collected from the LDLr^−/−^β-Gal^SM22^ mice were stained using a β-galactosidase stain kit (Special Media, MilliporeSigma, USA) as recommended by the manufacturer to genetically fate map the aortic valve cells which once expressed SM22α. The X-gal-stained LDLr^−/−^β-Gal^SM22^ hearts were post-fixed with a modified Clark's fixative prior to processing and embedding in paraffin. The hearts collected from the LDLr^−/−^ApoB^100^Runx2^f/f^ and LDLr^−/−^ApoB^100^Runx2^ΔSMA^ mice were washed in cold phosphate-buffered saline (PBS) and fixed in 4% paraformaldehyde (PFA) for ~4 h. They were switched to 20% sucrose solution in the PBS at 4C overnight, followed by being embedded in OCT compound (Tissue Tek, USA) in the cryo molds. Serial sections were made in 5 and 10 μm thickness for the paraffin and cryo embedded tissue samples, respectively, and subject to various histochemical and immunohistochemical staining. For the osteosense labeling, the frozen section slides were warmed on the bench for 30 min, washed in PBS 2×, and incubated with osteosense (Perkin Elmer; diluted 1:50 in PBS) at 4°C overnight. The slides were then washed in PBS, counterstained with 6-diamidino-2-phenylindole (DAPI) (Invitrogen, Waltham, Massachusetts, United States), and mounted with a Prolong gold (Invitrogen) antifade mounting media.

The slides were labeled *via* immunofluorescence for Runx2. Briefly, the slides were warmed on the bench for 30 min, washed with PBS 2X, and incubated in cold methanol for 10 min. The slides were washed in PBS and antigen retrieval was performed using 10 mM citrate buffer. The slides were placed in a citrate buffer at 65°C for 45 min, cooled on the bench for 20 min, washed in cold water, and then in PBS. The autofluorescence was reduced by incubating the slides in 0.1% sodium borohydride for 10 min and washed in PBS followed by incubation in a blocking solution (10% normal donkey serum, 0.25% BSA in PBS) for 1 h at room temperature (RT). The sections were then incubated in a primary antibody solution (10% donkey serum, 0.1% BSA in PBS) containing Runx2 primary antibody (Novus, Rabbit polyclonal; 1:200) at 4°C overnight. The next day, the slides were washed in PBS, incubated in a 1:500 Donkey anti rabbit Cy3 (Vector) for 30 min at RT in the dark. The slides were then washed in PBS, counterstained with DAPI, and mounted with a Prolong gold antifade mounting media.

### Imaging and Quantification

Fluorescent microscopy was performed using a Nikon Eclipse E800 (Nikon, Minato City, Tokyo, Japan) with a 20× objective. Fluorescence was detected using a Rhodamine Red filter cube (Runx2) and Texas Red (far red) filter cube for osteosense. All the images were captured with the Nikon NIS elements v3.1 software. The color intensity was measured using the NIS elements v3.1 software and expressed as a percentage of the total areas. A total of three aortic valve cross-sections of 100 × 300 μm were used for the imaging per animal. For quantification purposes, the aortic valve was divided into the sinus wall and the leaflet (41). The leaflet was further divided into the hinge (base of leaflet) and the cusp region.

### Quantitative Real-Time Polymerase Chain Reaction

The total RNA was extracted from the aortic valves (sinus wall, leaflet hinge, and leaflet cusps) pooled from 4 to 10 mice using a RNeasy mini kit. RNase- free DNase I (Qiagen, USA) was used to digest the contaminating DNA. The total RNA (0.5 to 1 μg) was used to synthesize the first-strand complementary DNA (cDNA) using Omniscript (Qiagen) at 37°C for 1 h. The cDNA was used to determine the expression of Runx2, OCN, ALP, OPN, Collagen X (ColX), and MMP13 using a TaqMan quantitative real-time PCR (qPCR). The primer/probe sequences used in the study are listed in Table 1. The primer/probe sets for ColX (Mm00487041_m1) and MMP13 (Mm00439491_m1) were purchased from Life Technologies (Carlsbad, CA, United States), with the assay ID listed in parentheses (40). The gene expression levels were normalized to 18s ribosomal RNA levels and expressed as a fold of the control samples.

**Table 1:** Table of primers.

**Gene**	**Forward primer Sequence (5′ – 3′)**	**Reverse primer Sequence (3′ – 5′)**	**Probe Sequence (5′ – 3′)**
Alp	CAAGGACATCGCATATCAGCTAA	CAGTTCTGTTCTTCGGGTACATGT	FAM-AGGATATCGACGTGATCAT-MGB
OCN	CTGGCTGCGCTCTGTCTCT	GACATGAAGGCTTTGTCAGACTCA	FAM-TGACCTCACAGATGCCAA-MGB
OPN	TGAGGTCAAAGTCTAGGAGTTTCC	TTAGACTCACCGCTCTTCATGTG	FAM-TTCTGATGAACAGTATCCTG-MGB
Runx2	CACCGACAGTCCCAACTTCCT	ACGGTAACCACAGTCCCATCTG	FAM-CCTTCAAGGTTGTAGCCCT-MGB

### Statistics

Statistical analysis was performed using Prism (GraphPad, USA). Data are shown as means ± SD. A Shapiro-Wilk test was performed to assess the normal distribution of data samples. For the data that were normally distributed, a Student's *t*-test was used for the comparison of the two groups, and for the data not normally distributed, Mann-Whitney non-parametric tests were used. One-way ANOVA with Bonferroni's test for was used for the comparison of multiple groups.

## Results

### Runx2+ VICs in Diseased Aortic Valves of Diabetic LDLr^–/–^β-Gal^SM22^ Mice Are Derived From SM22+ Precursors

**Figure 1:**
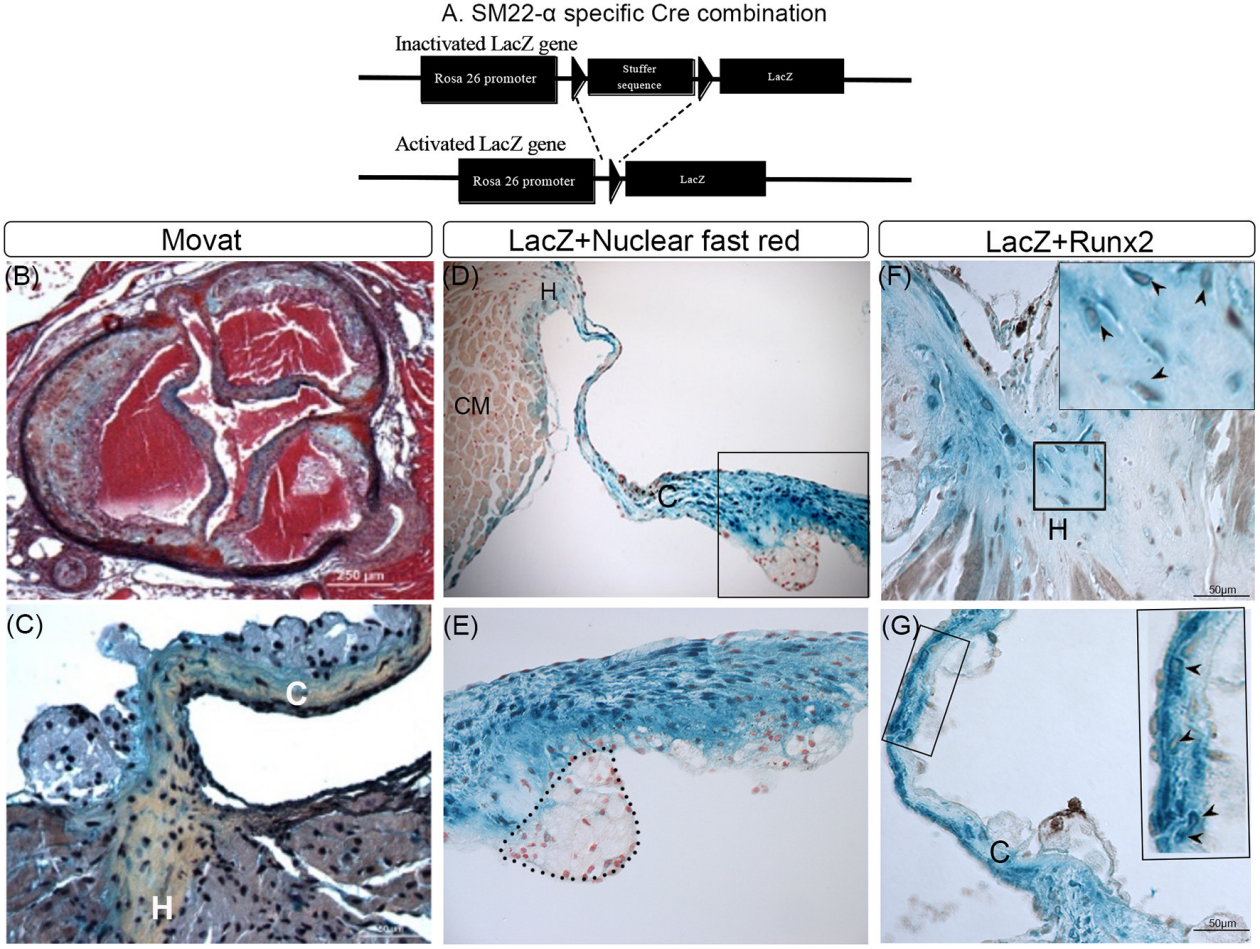
Lineage tracing of runt-related transcription factor 2 (Runx2)+ valvular interstitial cells (VICs) and sinus wall cells. **(A)** Schematic showing how Cre recombination leads to a permanent activation of the LacZ gene in SM22α+ VICs. **(B–G)** LDLr^−/−^:β-Gal^SM22^ mice were fed a diabetogenic (T2DM) diet for 28 weeks. Cartilaginous matrices (yellow collagen staining) in aortic valves were determined by Movat pentachrome staining **(B,C)**. VICs and sinus wall cells were stained blue with X-gal **(D–G)**. **(D,E)** Cell nuclei were counterstained with nuclear fast red. Note macrophage foam cells in the aortic side of the thickened valve leaflet that was not stained by X-gal (dashed line; **D,E**). CM, cardiomyocyte cells **(E)**. Higher magnification (60x) image of boxed area in **(D)**. Immunohistochemical staining of X-gal stained (blue) hinge **(F)** and cusps **(G)** of the aortic valve for Runx2 (brown, arrowheads). Inset in **(F,G)** are enlarged images of the boxed area. C, Cusp; H, Hinge.

Activated VICs are increased in CAVD and distinguished from quiescent VICs by the expression of several myofibroblast markers, including SM22α and SMA (19, 42). It has been suggested, but not proven, that Runx2 expressing osteochondrogenic cells that develop in diseased aortic valve leaflets are derived from SM22α+ VICs. To prove this, lineage tracing analyses in LDLr^−/−^β-Gal^SM22^ mice were performed. As shown in Figure 1A, the cell-specific Cre recombination in LDLr^−/−^β-Gal^SM22^ leads to a permanent expression of the LacZ gene in the nucleus of the cells derived from the SM22α+ precursors. Following T2DM diet feeding, the LDLr^−/−^β-Gal^SM22^ mice developed valve inflammation, sclerosis, leaflet thickening, and sinus wall lesions as shown by the Movat pentachrome staining (Figure 1B). Furthermore, many cells within the leaflet hinge and cusp regions showed a chondrocyte-like morphology and were surrounded by proteoglycan and collagen-rich matrices (arrows, Figure 1C). Importantly, many cells within the valve leaflet cusp and hinge areas were stained blue by X-gal (Figures 1D–G), identifying them as being originally derived from the SM22α+ VICs. As expected, the sinus wall cells were also stained blue with Xgal, consistent with the smooth muscle cell (SMC) origin. Finally, the chondrocyte-like, X-gal-positive cells in the leaflet hinge (Figure 1F, arrowheads) and cusp (Figure 1G, arrowheads) areas co-expressed Runx2. Approximately 45 and 52% of the Runx2+ cells were Xgal+ in the aortic leaflet hinge and cusp, respectively. The Xgal+Runx2+ cells were also observed in the sinus wall lesions but not in the underlying aortic root SMCs (data not shown), consistent with previous observations in the atherosclerotic aorta (33, 35). In contrast, the inflammatory cells were X-gal and Runx2 negative (area enclosed by a dashed line, Figure 1D). The Runx2+ and Xgal-ve cells were noted in the valve hinge and cusp lumenal areas (Figures 1F,G). These Runx2+ cells were likely derived from the non-SM22α+ VIC precursors, including quiescent VIC, endothelial cells *via* EndoMT, and/or circulating precursors. Consistent with this, recent studies have reported that valve endothelium can express Runx2 (43).

### Conditional Deficiency of Runx2 in aVICs and Sinus Wall Cells Decreases Osteochondrogenic Differentiation in Diseased LDLr^–/–^ Mouse Aortic Valves

**Figure 2:**
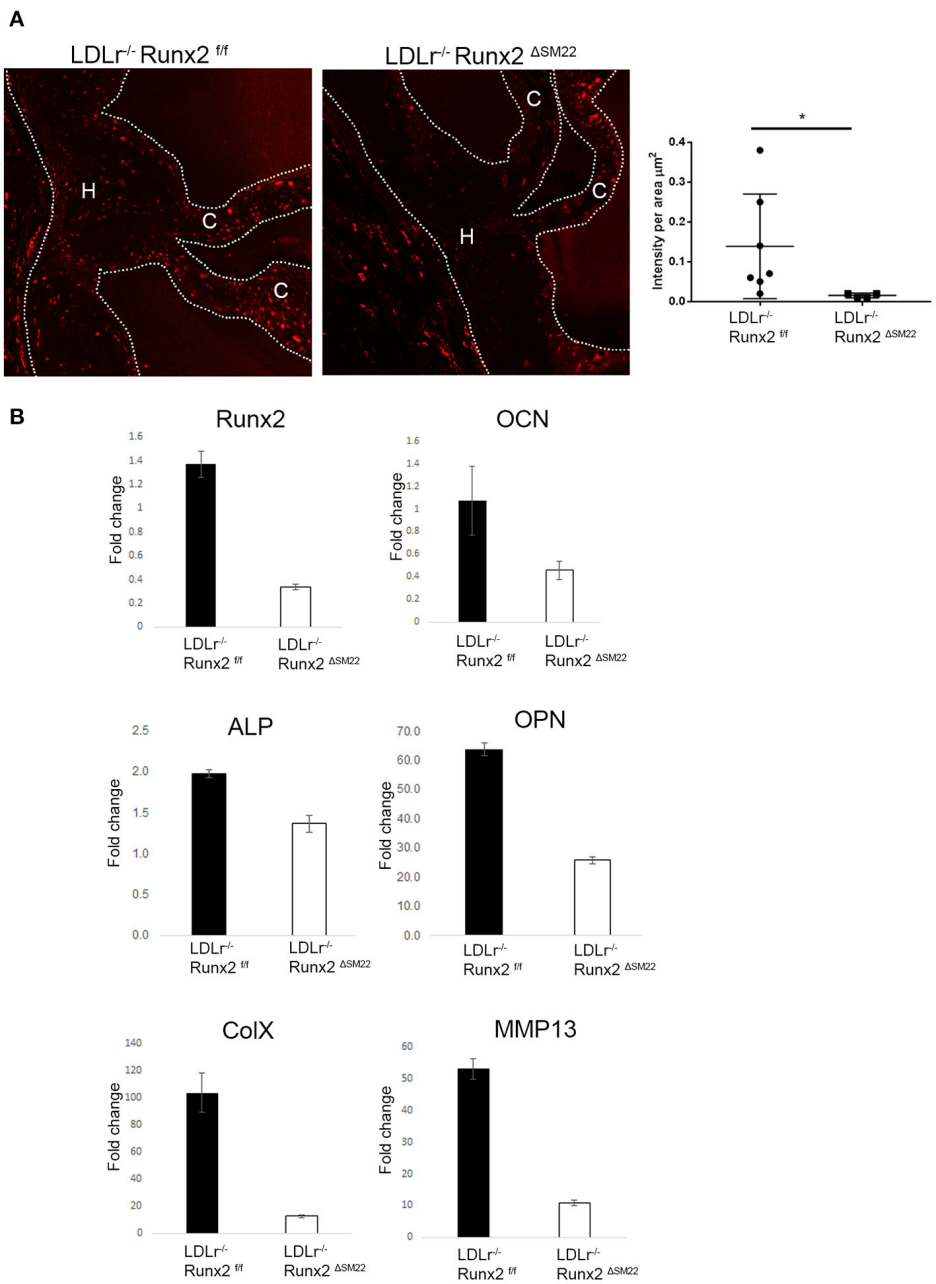
Runx2 in SM22α+ VICs and sinus wall cells contributes to osteochondrogenic differentiation in the aortic valve. **(A)** LDLr^−/−^:Runx2^ΔSM22^ mice and LDLr^−/−^:Runx2^f/f^ littermates were fed with a T2DM diet for 26 weeks. Aortic valves (dashed lines) were collected for Runx2 immunofluorescent staining. Graphs show the quantification of Runx2 intensity in leaflets (cusp + hinge) normalized to the area. **(B)** LDLr^−/−^:Runx2^f/f^ and LDLr^−/−^:Runx2^ΔSM22^ mice were fed with T2DM for 26 weeks. Four to ten aortic valves were pooled to generate RNA. Runt-related transcription factor 2, osteocalcin (OCN), alkaline phosphatase (ALP), osteopontin (OPN), and chondrocyte markers, Collagen X (ColX), and MMP13, gene expression was determined in triplicate *via* real-time quantitative PCR (qRT-PCR). Differences between groups were analyzed by student's *t*-test. *p < 0.05. Data shown are mean ± SD. C, Cusp; H, Hinge.

To determine whether Runx2 regulates osteochondrogenic differentiation in diabetic LDLr^−/−^ mice, Runx2 was selectively deleted in the aVICs and sinus wall cells. The LDLr^−/−^:Runx2^ΔSM22^ and LDLr^−/−^:Runx2^f/f^ control littermates were fed with the T2DM diet for 26 weeks. The SM22αCre-directed removal of Runx2 in the aortic valves of the LDLr^−/−^:Runx2^ΔSM22^ mice was efficient. Abundant Runx2 localized to the nucleus was observed in the aVICs and sinus wall cells of the LDLr^−/−^:Runx2^f/f^ mice fed with the T2DM diet for 26 weeks (Supplementary Figure 1). As shown in Figure 2A, the Runx2 immunostaining was largely reduced in the valve of the LDLr^−/−^:Runx2^ΔSM22^ mice compared with the LDLr^−/−^:Runx2^f/f^ mice. A qRT-PCR using the mRNA extracted from pooled aortic valve specimens confirmed the immunostaining results (Figure 2B). Further, the qRT-PCR analysis confirmed that the depletion of Runx2 in the SM22α+ aVICs and sinus wall cells resulted in a dramatically blocked osteochondrogenic maturation as indicated by the markedly reduced levels of ALP, OPN, OCN, ColX, and MMP13 mRNA, (Figure 2B). These data confirm an important role for Runx2 in driving osteochondrogenic differentiation in the diseased aortic valve.

### Development of a Spatiotemporal, Inducible Mouse Model to Transiently Deplete Runx2 Early in CAVD

While the LDLr^−/−^ mice fed with a diabetogenic diet developed aortic valves with sclerotic and osteochondrogenic changes, they displayed sparse calcification, a key feature of human CAVD (44, 45). Perhaps consequently, the LDLr^−/−^ mice did not develop valve dysfunction consistent with the clinically significant AS in humans (46). To assess the role of Runx2 in aortic valve calcification and function, we utilized the LDLr^−/−^:ApoB^100^ mice fed with the T2DM diet (39). As we have previously published, these mice developed a high frequency of moderate to severe AS, ventricular dysfunction, matrix disorganization, and most importantly, valve calcification (39). Furthermore, the availability of an inducible SMA Cre allowed us to transiently deplete Runx2 specifically from the aVICs and sinus wall cells at an early stage of the disease, thus allowing us to determine whether Runx2 was required for CAVD progression.

**Figure 3:**
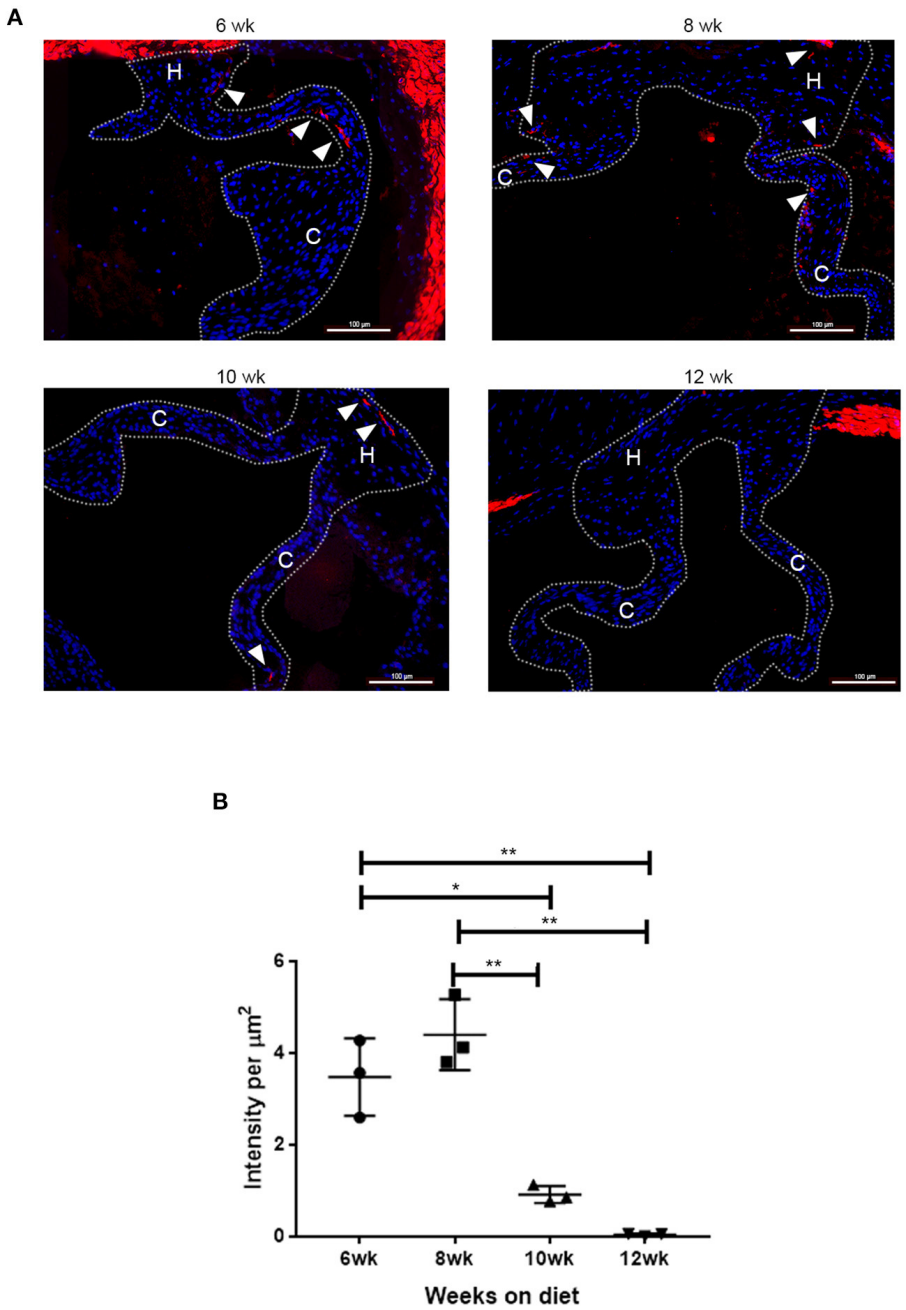
Time course of *de novo* smooth muscle α-actin (SMA) expression in disease aortic valves. **(A)** Aortic valve sections from LDLr^−/−^ApoB^100^ mice on the T2DM diet labeled for SMA (red) and 6-diamidino-2-phenylindole (DAPI) (blue). Cusp and hinge regions are outlined with dashed lines. The SMA expression is observed in the valve leaflets in sections from 6 weeks through 10 weeks of the T2DM diet mice (white arrowheads). **(B)** Quantification of fluorescence intensity in SMA labeled sections from LDLr^−/−^ApoB^100^ mice on the T2DM diet for 6–12 weeks (*n* = 3 per time point). Differences between groups were analyzed by one-way ANOVA with Bonferroni's correction. *p < 0.05, **p < 0.005.

To determine the time frame for the spatiotemporal Runx2 deletion, the LDLr^−/−^:ApoB^100^ mice were fed with the T2DM diet and the time course for the appearance of SMA+ cells in the aortic valves was determined. As shown in Figure 3, *de novo* SMA expression was observed in cells of the T2DM LDLr^−/−^:ApoB^100^ mice aortic valves as early as 6 weeks on diet. The SMA+ aVICs were found in the leaflet cusp and hinge regions. In addition, *de novo* expression of SMA was observed in the valve sinus wall lesions. At 8 and 10 weeks on the T2DM diet, the SMA+ cells were significantly decreased and disappeared by 12 weeks, consistent with the early and transient appearance of SMA+ cells during CAVD development. Smooth muscle α-actin was also strongly expressed in the aortic root wall cells at all time points observed, consistent with the expression in the SMCs. No SMA staining was observed in the healthy valve leaflets of the C57BL6 wild-type mice [data not shown and (47)].

**Figure 4:**
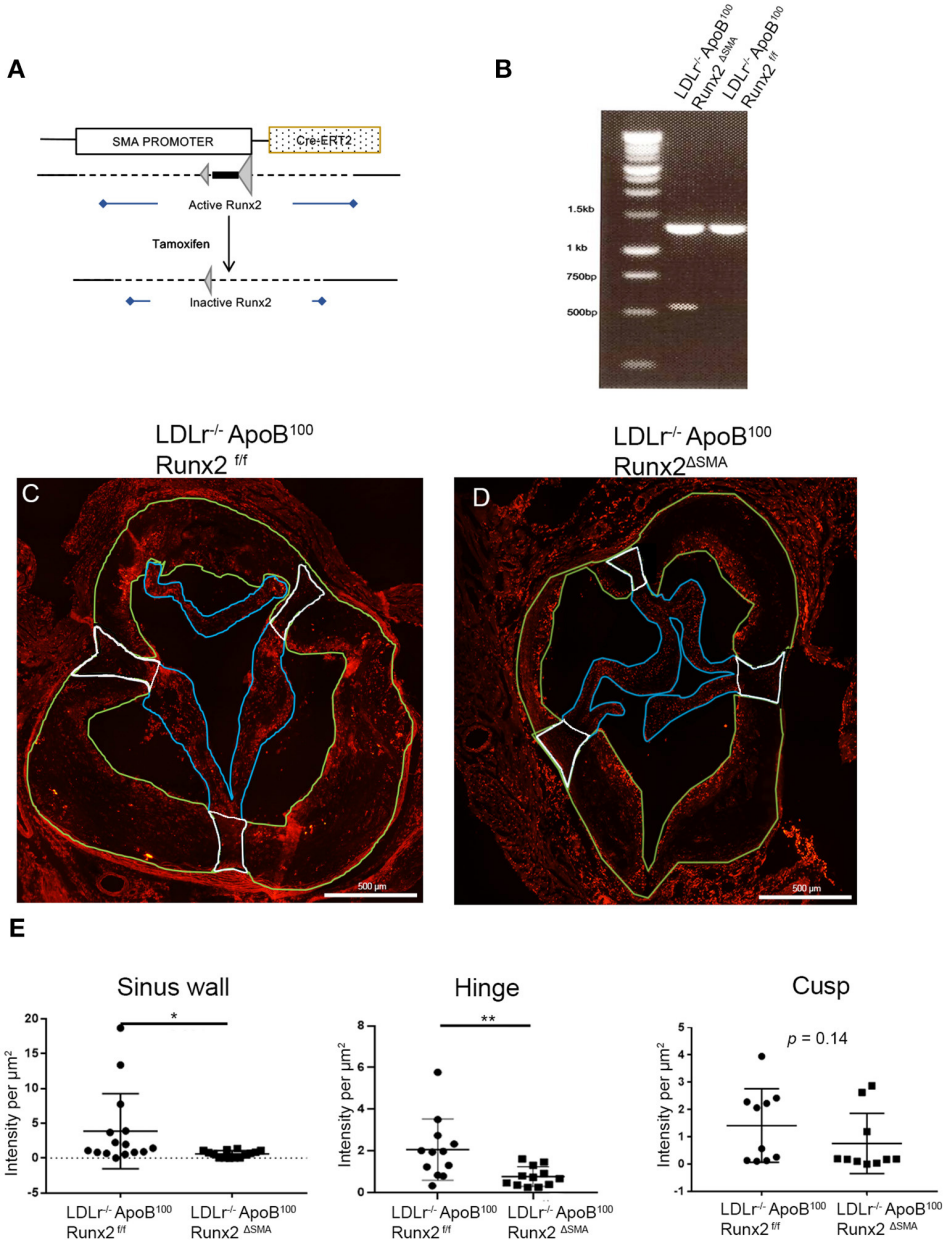
Runx2 depletion in the aortic valve. **(A)** Schematic tamoxifen-induced Cre recombination that leads to a temporal inactivation of Runx2 in SMA+ VICs and sinus valve cells. **(B)** PCR genotyping to amplify the loxP flanked sites to confirm Runx2 depletion in the aortic valve. IF of aortic valve sections from LDLr^−/−^ApoB^100^Runx2^f/f^ **(C)** and LDLr^−/−^ApoB^100^Runx2^ΔSMA^ **(D)** mice for Runx2. Green borders highlight the sinus wall region, white borders highlight the hinge region and blue borders highlight the cusp region. **(E)** Quantification of Runx2 intensity in valve sections in the sinus wall, hinge, and cusp regions (*n* = 15 per group). Differences between groups were analyzed by Mann-Whitney non-parametric tests. *p < 0.05, **p < 0.005. Data shown are mean ± SD, *n* = 15.

Based on the time-course of *de novo* SMA expression in the diseased valve leaflet and sinus wall cells, we generated a CAVD model with a spatiotemporally controlled depletion of Runx2. Tamoxifen inducible Cre (CRE-ERT2) controlled by the SMA promoter in the LDLr^−/−^ApoB^100^ was used to create the LDLr^−/−^ApoB^100^Runx2^f/f^: SMACreERT2^+/o^ (LDLr^−/−^ApoB^100^Runx2^ΔSMA^) mouse line as shown in Figure 4A. The LDLr^−/−^ApoB^100^Runx2^ΔSMA^ and control LDLr^−/−^ApoB^100^Runx2^f/f^ mice on the T2DM diet were treated with tamoxifen from week 6 through 8 to induce Cre activation and Runx2 depletion specifically in the aVICs and sinus wall cells expressing SMA.

The targeted depletion of Runx2 in the aortic valve was confirmed by DNA genotyping (Figure 4B). Recombined allele (527 bp band) was observed in the LDLr^−/−^ApoB^100^Runx2^ΔSMA^ but not LDLr^−/−^ApoB^100^Runx2^f/f^ valve tissue. Recombination was also observed in the positive control tissues, aortic arch, and bladder, but not in the negative control tissues, liver, and trachea in the samples obtained from the LDLr^−/−^ApoB^100^Runx2^ΔSMA^ mice (Supplementary Figure 2). Finally, the aortic valve sections from the LDLr^−/−^ApoB^100^Runx2^f/f^ and LDLr^−/−^ApoB^100^Runx2^ΔSMA^ were stained for Runx2 protein *via* immunofluorescence (Figures 4C,D). Quantification of the images demonstrated a significant decrease in the Runx2 expression in both the aortic valve leaflet hinge and sinus wall lesions of the LDLr^−/−^ApoB^100^Runx2^ΔSMA^ compared with the LDLr^−/−^ApoB^100^Runx2^f/f^ valves (Figure 4E; *n* = 15 per group). A decreasing trend was noted in the valve cusps of LDLr^−/−^ApoB^100^Runx2^ΔSMA^ compared with that of the LDLr^−/−^ApoB^100^Runx2^f/f^ valves, but this did not attain statistical significance. No Runx2 expression was observed in the aortic root wall SMCs in either group (data not shown).

### Transient, Inducible Runx2 Depletion Improves Aortic Valve Function in the LDLr^–/–^ApoB^100^Runx2^Δ*SMA*^ Mouse Model

**Figure 5:**
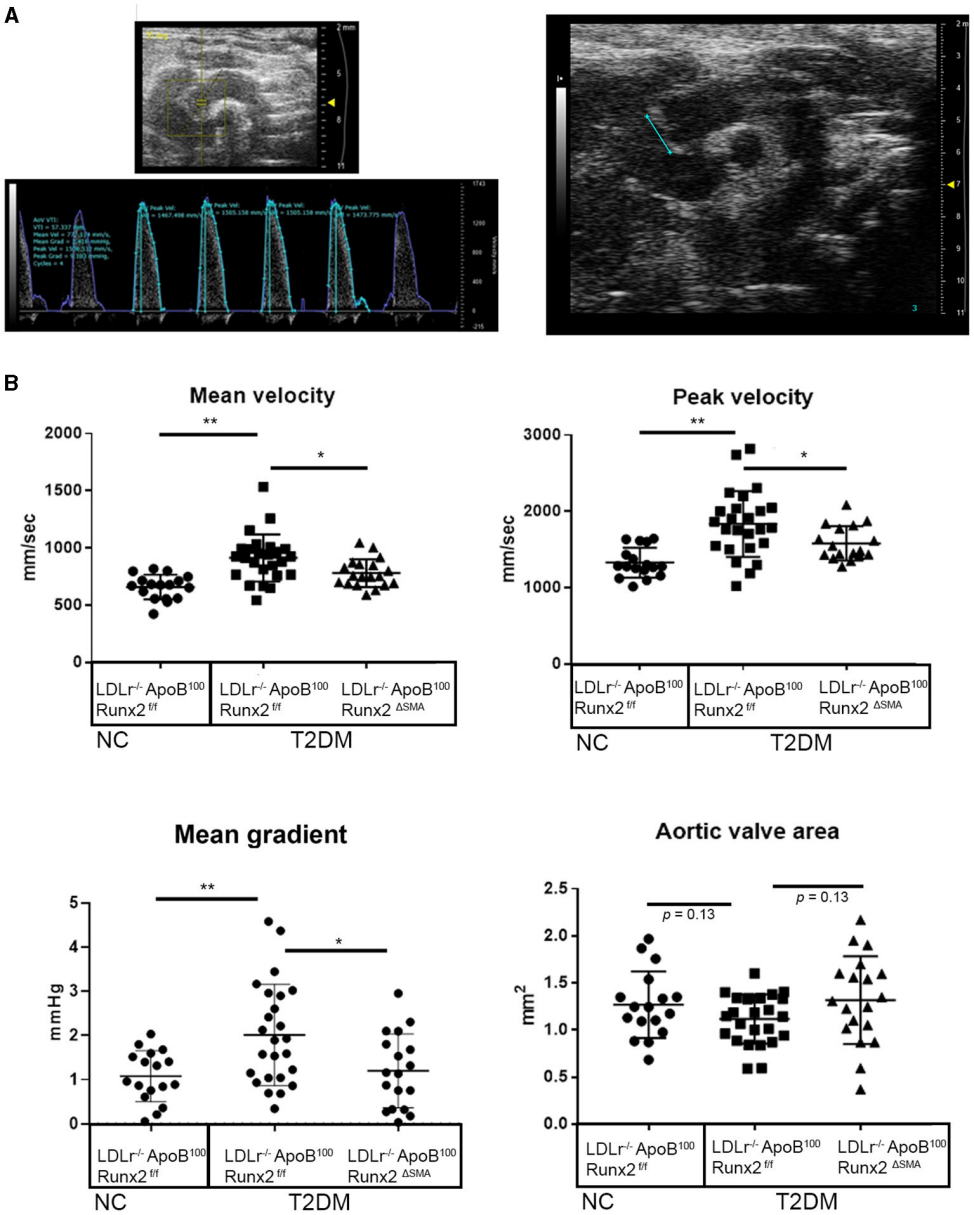
Runx2 depletion in SMA+ VICs and sinus walls improves aortic valve function. Representative image of aortic velocity (AoV) flow measurements for blood flow parameters and B mode image used for aortic valve area measurements **(A)**. **(B)** Quantitative analysis of the mean velocity, AoV peak velocity, mean gradient, peak gradient, and aortic valve area between the 26 wk LDLr^−/−^ApoB^100^Runx2^f/f^ normal chow (NC, *n* = 18), T2DM diet LDLr^−/−^ApoB^100^Runx2^f/f^ (*n* = 25), and T2DM diet LDLr^−/−^ApoB^100^Runx2^ΔSMA^(*n* = 20). The differences between groups were analyzed by one-way ANOVA with Bonferroni's correction *p < 0.05, **p < 0.005. Data shown are mean ± SD.

**Figure 6:**
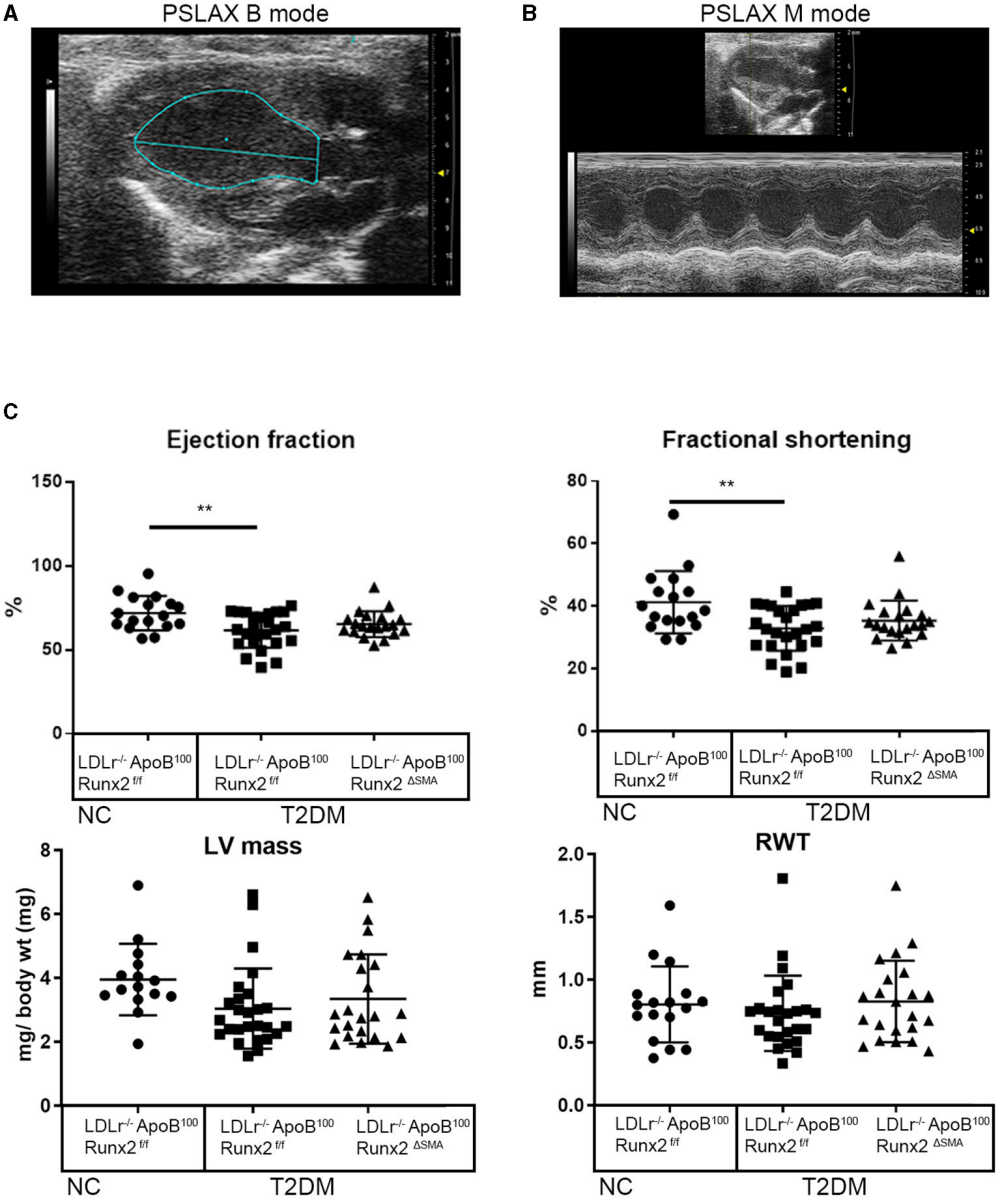
SMA-specific depletion of Runx2 had no significant effect on ventricular function. Representative images of long-axis B-mode **(A)** and M-mode **(B)** images were used for the assessment of the left ventricular function. **(C)** Quantitative analysis of ejection fraction, fractional shortening, left ventricle mass (normalized to body weight), and relative wall thickness (RWT), between the 26 weeks LDLr^−/−^ApoB^100^Runx2^f/f^ normal chow (NC, *n* = 18), T2DM diet LDLr^−/−^ApoB^100^Runx2^f/f^ (*n* = 25) and T2DM diet LDL*r*^−/−^ApoB^100^Runx2^ΔSMA^ (*n* = 20) groups. The differences between groups were analyzed by one-way ANOVA with Bonferroni's correction **p < 0.005. Data shown are mean ± SD.

To determine whether the transient depletion of Runx2 in the SMA+ aVICs and sinus wall cells improved the aortic valve hemodynamic function, we performed an echocardiographic analysis of the LDLr^−/−^ApoB^100^Runx2^ΔSMA^ (*n* = 20) and LDLr^−/−^ApoB^100^Runx2^f/f^ (*n* = 25) mice after 26 weeks on the T2DM diet (*n* = 20) and LDLr^−/−^ApoB^100^Runx2^f/f^ on NC diet (*n* =18). The tissue Doppler imaging showed that Runx2 depletion improved aortic valve function, as evidenced by a significantly reduced aortic valve peak velocity (~10%, *p* < 0.05), mean velocity (~10%, *p* < 0.05) and mean gradient (~50%, *p* < 0.05) in the LDLr^−/−^ApoB^100^Runx2^ΔSMA^ mice in comparison with the LDLr^−/−^ApoB^100^Runx2^f/f^ mice (Figure 5). The calculated AVA, as determined by the continuity equation, showed a trend toward improvement in the T2DM fed LDLr^−/−^ApoB^100^Runx2^ΔSMA^ mice (~20%) compared with LDLr^−/−^ApoB^100^Runx2^f/f^ (*p* = 0.13) However, no statistically significant difference was observed between the three groups (Figure 5). Cardiac muscle function parameters as determined by the ejection fraction (EF) and fractional shortening (FS) were significantly lower in the LDLr^−/−^ApoB^100^Runx2^f/f^ mice when compared with the NC group, however, appeared to be within normal parameters [EF > 60% and FS > 30% (48, 49)] in all groups (Figure 6). The left ventricle mass (normalized to body weight) and the relative wall thickness (RWT) were not different between the three groups (Figure 6). These data suggest that Runx2 depletion in the SMA+ aVICs and sinus wall cells early in the disease blocks further disease progression, leading to some improvement in aortic valve function without changes in heart function in mice.

### Runx2 Depletion Reduced Calcification in the Aortic Valve of LDLr^–/–^ApoB^100^Runx2^Δ*SMA*^ Mice

**Figure 7:**
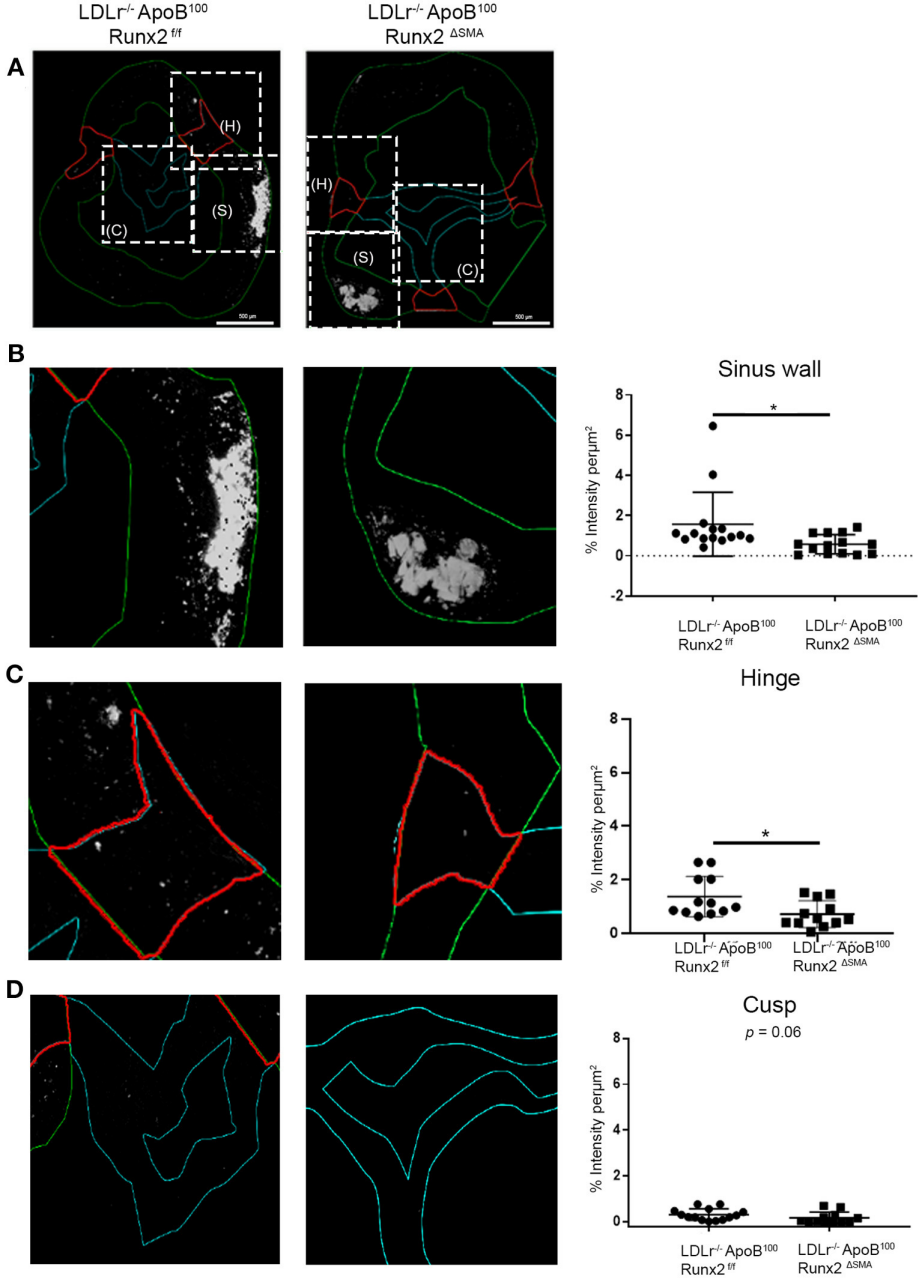
Runx2 depletion decreased calcification in the aortic valve. **(A)** Representative images of valve sections labeled with osteosense from the LDLr^−/−^ApoB^100^Runx2^f/f^ and LDLr^−/−^ApoB^100^Runx2^ΔSMA^ mice to assess calcification. Boxed areas (dashed lines) within **(A)** represent the sinus wall (green), hinge (red), and cusp (teal). Higher magnification of boxed area from **(A)**, representing the sinus wall **(B)**, hinge **(C)**, and cusp **(D)** in the aortic valve and their respective intensity quantification graphs (*n* = 15 per group). The differences between groups were analyzed by Mann-Whitney non-parametric tests. *p < 0.05. Data shown are mean ± SD.

To understand the mechanisms by which transient Runx2 depletion in the aVICs and SMA+ sinus wall cells improved valve function, the aortic valve sections were examined for valve leaflet thickness, cross-sectional area, and calcification. No significant differences in valve leaflet (hinge and cusp) thickness or valve cross-sectional area were observed by the Movat staining (Supplementary Figure 3). To examine the valve calcification, the aortic valve sections were labeled with osteosense (Figure 7). Aortic valve images were divided into the sinus wall (Figure 7B), leaflet hinge (Figure 7C), and leaflet cusp (Figure 7D), and the intensity of the Osteosense label was measured as a percentage of the respective areas (*n* = 15 per group). The depletion of Runx2 led to a statistically significant ~50% decrease in calcification in both the valve sinus wall and leaflet hinge region of the LDLr^−/−^ApoB^100^Runx2^ΔSMA^ when compared with the LDLr^−/−^ApoB^100^Runx2^f/f^ 26 weeks on the T2DM diet mice (Figures 7B,C, respectively). While the aortic valve cusp also showed a trend of ~40% decrease (*p* = 0.06) in calcification between these mice, the difference did not attain statistical significance (Figure 7D).

## Discussion

Our findings showed, for the first time, that many Runx2 expressing osteochondrogenic cells in the aortic valve leaflet cusp and hinge are derived from the SM22α+ aVICs and sinus wall cells in diseased LDLr^−/−^ aortic valves. Further, we showed that the constitutive depletion of Runx2 in the SM22α+ cells in LDLr^−/−^:Runx2^ΔSM22^ mice inhibited osteochondrogenic differentiation. Finally, we assessed the role of spatiotemporal Runx2 depletion in valve cells at an early stage of CAVD in LDLr^−/−^ApoB^100^ mice fed with a diabetogenic diet. Using this model, we showed that the transient depletion of Runx2 in the aVICs and sinus wall cells resulted in the normalization of the aortic peak velocity, mean velocity, and mean gradient but did not affect the left ventricular functional parameters. Furthermore, Runx2 depletion reduced calcification in the aortic valves. These results suggest that Runx2 expressing cells play a significant role in promoting osteochondrogenic differentiation and aortic valve calcification, which ultimately contributes to hemodynamic dysfunction in CAVD.

Activated VICs, defined as valve cells that express myofibroblast-like markers including SMA and SM22α, are commonly observed in human and mouse aortic valve disease. While both SMA and SM22α are expressed in SMC, a component of human aortic valve leaflets (19) and human and mouse sinus walls, undiseased adult mouse aortic valve leaflets contain few to no VICs expressing smooth muscle markers (50). Using cell lineage studies, we demonstrated for the first time that many cells present in the diseased mouse aortic valve leaflets are derived from SM22α+ VICs, and that a subset of these cells expressed Runx2. Furthermore, the depletion of Runx2 in the SM22α+ aVICs and sinus wall cells decreased the mRNA expression of osteochondrogenic markers, ALP, OPN, OCN, ColX, and MMP13 in the diabetic LDLr^−/−^ Runx2^ΔSM22^ compared with the diabetic LDLr^−/−^Runx2^f/f^ mice aortic valve tissue. Thus, the Runx2 in the SM22+ aVICs and sinus wall cells drives osteochondrogenesis early in valve disease. Consistent with these findings, Runx2 is a master regulator of skeletal chondrocyte and osteoblast differentiation and has been shown to promote osteochondrogenic differentiation of SMCs during vascular calcification (40, 51–53). While the present studies highlight the importance of SM22+ aVICs as precursors for osteochondrogenic cells in the aortic valve leaflets, our data also show that cells that are SM22 negative but Runx2 positive are also present in diseased mouse valves, including quiescent VIC, valvular endothelial cells, and circulating macrophages (54–56), and may contribute to osteochondrogenic changes.

Given the decrease in the osteochondrogenic differentiation observed in the LDLr^−/−^ Runx2^ΔSM22^, we postulated that Runx2 depletion in myofibroblast-like aVICs and sinus wall cells would block disease progression and ameliorate AS associated with CAVD. To test this, we utilized LDLr^−/−^ApoB^100^ mice fed with a T2DM diet. In contrast to diabetic LDLr^−/−^ mice, LDLr^−/−^ApoB^100^ mice fed with a T2DM diet develop advanced CAVD, including ECHO-detectable hemodynamic dysfunction at very high frequency. (39, 57). Furthermore, the appearance of SMA expressing aVIC and sinus wall cells was transient and preceded the onset of AS. This allowed us to deplete Runx2 selectively in the SMA+ aVICs and sinus wall cells at a very early stage of CAVD to determine its role in CAVD progression. Hemodynamically, we observed that transient Runx2 depletion resulted in significant decreases in the aortic peak velocity and peak gradient, as well as mean velocity and mean gradient of the LDLr^−/−^ApoB^100^Runx2^ΔSMA^ compared with the control LDLr^−/−^ApoB^100^Runx2^f/f^ mice. In humans, a two-fold change in the mean gradient from 10 to 20 mmHg is clinically defined as a moderate disease (46). In our model, age-matched LDLr^−/−^ApoB^100^ mice fed with a normal chow diet had a mean gradient of <1 mmHg. In contrast, the diseased LDLr^−/−^ApoB^100^ mice fed with a diabetogenic diet had a mean gradient of about 2 mmHg. This two-fold increase in the mean gradient suggests the progression of CAVD following diabetogenic diet feeding. The temporal deletion of Runx2 in SMA+ cells in mice fed diabetogenic diet significantly decreased the mean gradient by about two-fold compared with the fl/fl controls, suggesting that Runx2 depletion blocked the progression of CAVD.

To determine the major mechanisms underlying the effect of Runx2 depletion on aortic valve function in mice, we examined the aortic valve thickness, static aortic valve diameter, and aortic valve calcification. We observed a significant decrease in the calcification in the aortic valve hinge and sinus wall area of the LDLr^−/−^ApoB^100^Runx2^ΔSMA^ mice compared with the control LDLr^−/−^ApoB^100^Runx2^f/f^ mice. On the other hand, no significant change in the valve leaflet thickness or aortic valve diameter between the groups was observed. A decreasing trend was observed for the leaflet cusp calcification, but this did not attain statistical significance. This was likely caused by the failure to significantly deplete Runx2 in the valve leaflet cusps in the LDLr^−/−^ApoB^100^Runx2^ΔSMA^ due to the presence of valve cells other than aVICs that were found to express Runx2. Our findings are consistent with several recent studies showing a positive correlation between Runx2 expression and calcification in aortic valves in people and animal studies (22, 38, 58). Our studies also suggest that leaflet hinge and sinus wall calcification may be sufficient to cause significant valve dysfunction in mice. The leaflet hinge and sinus wall were noted as major sites of mineralization observed in other murine CAVD studies (59–61).

While leaflet cusp calcification has been identified as a key culprit in clinical human AS (14), the roles of the valve leaflet hinge and sinus wall in subclinical aortic valve dysfunction in people have been less clear. Interestingly, Yutzey's group observed that calcification in human CAVD begins in the valve hinge region and precedes nodular leaflet calcification, which is typically observed in valves obtained from patients with clinical AS (62). Furthermore, a compliant sinus wall in the aortic root is required for several valve functions, including transport of blood with a minimal gradient between the left ventricle and the aorta, ensuring a wide flow variation, and preserving leaflet integrity (63). Calcification in the valve sinus wall would decrease the compliance and increase the stiffness of the aortic root, thereby potentially contributing to the valve dysfunction observed in our studies. Thus, it is possible that mild changes in valve function in people with non-clinical AS could be partly due to leaflet hinge and sinus wall calcification. Our findings that leaflet hinge and sinus wall calcification contribute to valve dysfunction even in the absence of extensive calcification of the valve leaflet cusps in mice support this notion.

In summary, we have shown that Runx2 expression in myofibroblast-like aVICS and sinus wall cells promotes osteochondrogenic differentiation and calcification in the mouse aortic valve leaflet hinge and sinus wall. Importantly, SMA+ aVIC and sinus wall lesion cells arise early and transiently in the disease process and Runx2 deficiency in these cells greatly diminishes the hemodynamic pathological manifestation of the disease. These studies suggest that the inhibition of Runx2 expression and/or its downstream signaling pathways may be useful as therapeutic strategies for CAVD.

## Data Availability Statement

The raw data supporting the conclusions of this article will be made available by the authors, without undue reservation.

## Ethics Statement

The animal study was reviewed and approved by University of Washington Institutional Animal Care and Use Committee.

## Author Contributions

SD and MSp worked out most of the experimental design. SD carried out most of the experiments, sample collection, and data analysis. MSp and CG devised the project and the main conceptual idea. SD, KP, and JL did the data collection. KP and JL assisted with the animal colony maintenance. EL helped with animal work and data collection. M-EL performed all experiments with the LDLr^−/−^ mice. MSc helped in interpretation of the ECHO and IF data. SD, MSp, MSc, and CG wrote the manuscript. CG supervised the project. All authors contributed to the article and approved the submitted version.

## Funding

This study was supported by the National Institutes of Health Grants HL139602, HL081785, and HL62329.

## Conflict of Interest

The authors declare that the research was conducted in the absence of any commercial or financial relationships that could be construed as a potential conflict of interest.

## Publisher's Note

All claims expressed in this article are solely those of the authors and do not necessarily represent those of their affiliated organizations, or those of the publisher, the editors and the reviewers. Any product that may be evaluated in this article, or claim that may be made by its manufacturer, is not guaranteed or endorsed by the publisher.

## Supplementary Material

Supplementary Figure 1 Nuclear localization of Runx2 in diseased valve leaflet and sinus wall cells. Sections of 26 weeks T2DM fed LDLr^−/−^Apob^100^Runx2^f/f^ mouse aortic valve labeled for Runx2 (red) and DAPI nuclear stain (blue). The merged image shows nuclear colocalization of Runx2 (pink/purple areas) in the sinus wall (S), hinge (H) and cusp (C) regions.

Supplementary Figure 2 Tissue specificity of Runx2 depletion in the Runx2^ΔSMA^ model. The PCR genotyping analysis of loxP flanked sites to confirm Runx2 depletion (presence of the 537 bp band) in DNA collected from the aortic arch (AA), bladder (B), liver (L), and trachea (T) of the LDLr^−/−^ApoB^100^Runx2^f/f^ and LDLr^−/−^ApoB^100^Runx2^ΔSMA^ mice.

Supplementary Figure 3 Runx2 depletion had no effect on valve cross-sectional area or leaflet area. **(A)** Representative images of the valve sections stained with Movat pentachrome in LDLr^−/−^ApoB^100^Runx2^f/f^ and LDLr^−/−^ApoB^100^Runx2^ΔSMA^ mice. **(B)** Graphs represent the quantification of valve cross-sectional area and leaflet area from Movat stained valve sections normalized to body weight. Differences between groups were analyzed by Mann-Whitney non-parametric tests. Data shown are mean ± SEM.
