# Supplementary figures and images for: Role of Runx2 in Calcific Aortic Valve Disease in Mouse Models
Source: Front Cardiovasc Med. 2021 Oct 29;8:687210. doi: 10.3389/fcvm.2021.687210 (PMC8585763; doi:10.3389/fcvm.2021.687210)

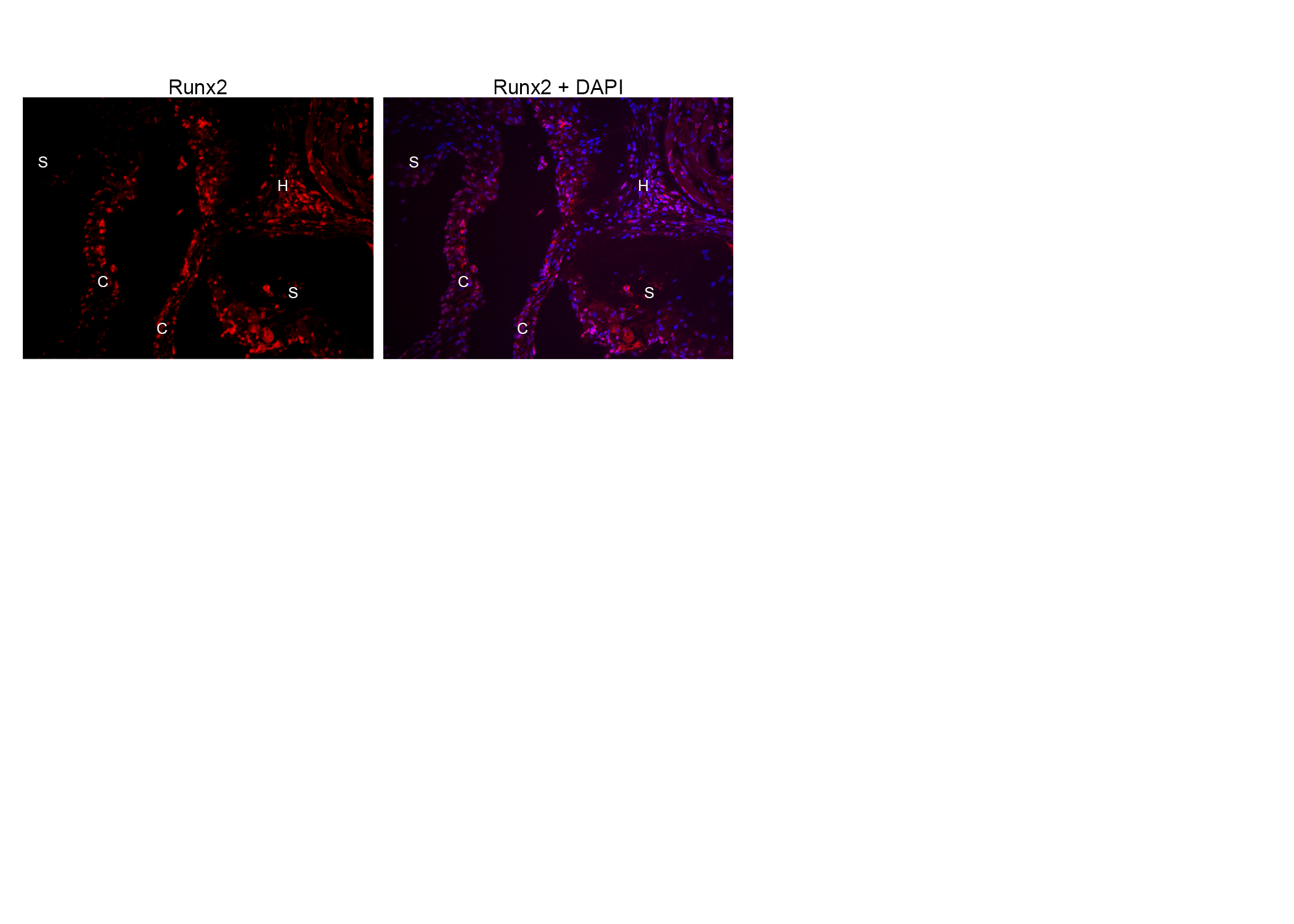

Supplement: Supplementary Figure 1 — Nuclear localization of Runx2 in diseased valve leaflet and sinus wall cells. Sections of 26 weeks T2DM fed LDLr−/−Apob100Runx2f/f mouse aortic valve labeled for Runx2 (red) and DAPI nuclear stain (blue). The merged image shows nuclear colocalization of Runx2 (pink/purple areas) in the sinus wall (S), hinge (H) and cusp (C) regions. [file Image_1.TIF]

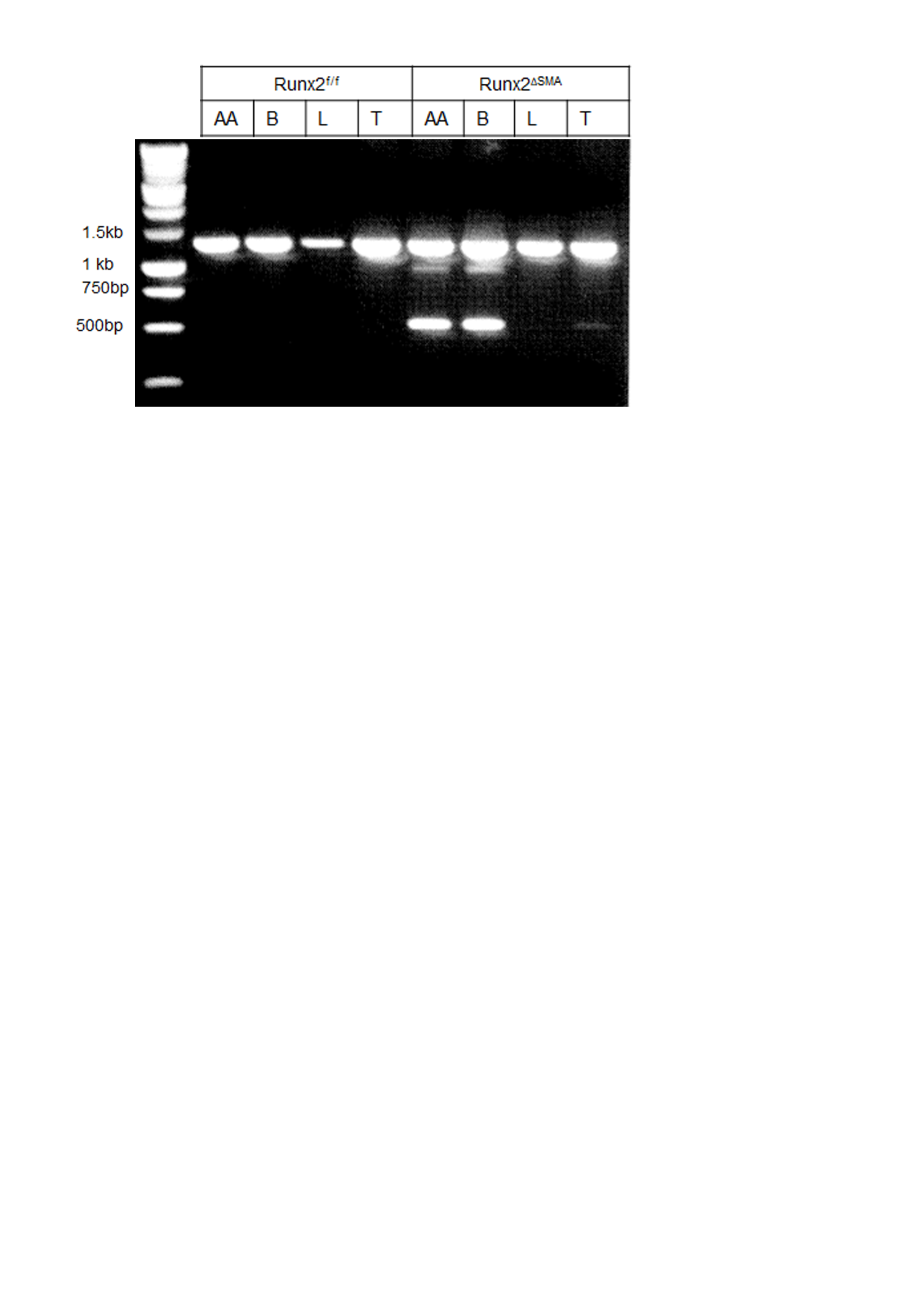

Supplement: Supplementary Figure 2 — Tissue specificity of Runx2 depletion in the Runx2ΔSMA model. The PCR genotyping analysis of loxP flanked sites to confirm Runx2 depletion (presence of the 537 bp band) in DNA collected from the aortic arch (AA), bladder (B), liver (L), and trachea (T) of the LDLr−/−ApoB100Runx2f/f and LDLr−/−ApoB100Runx2ΔSMA mice. [file Image_2.TIF]

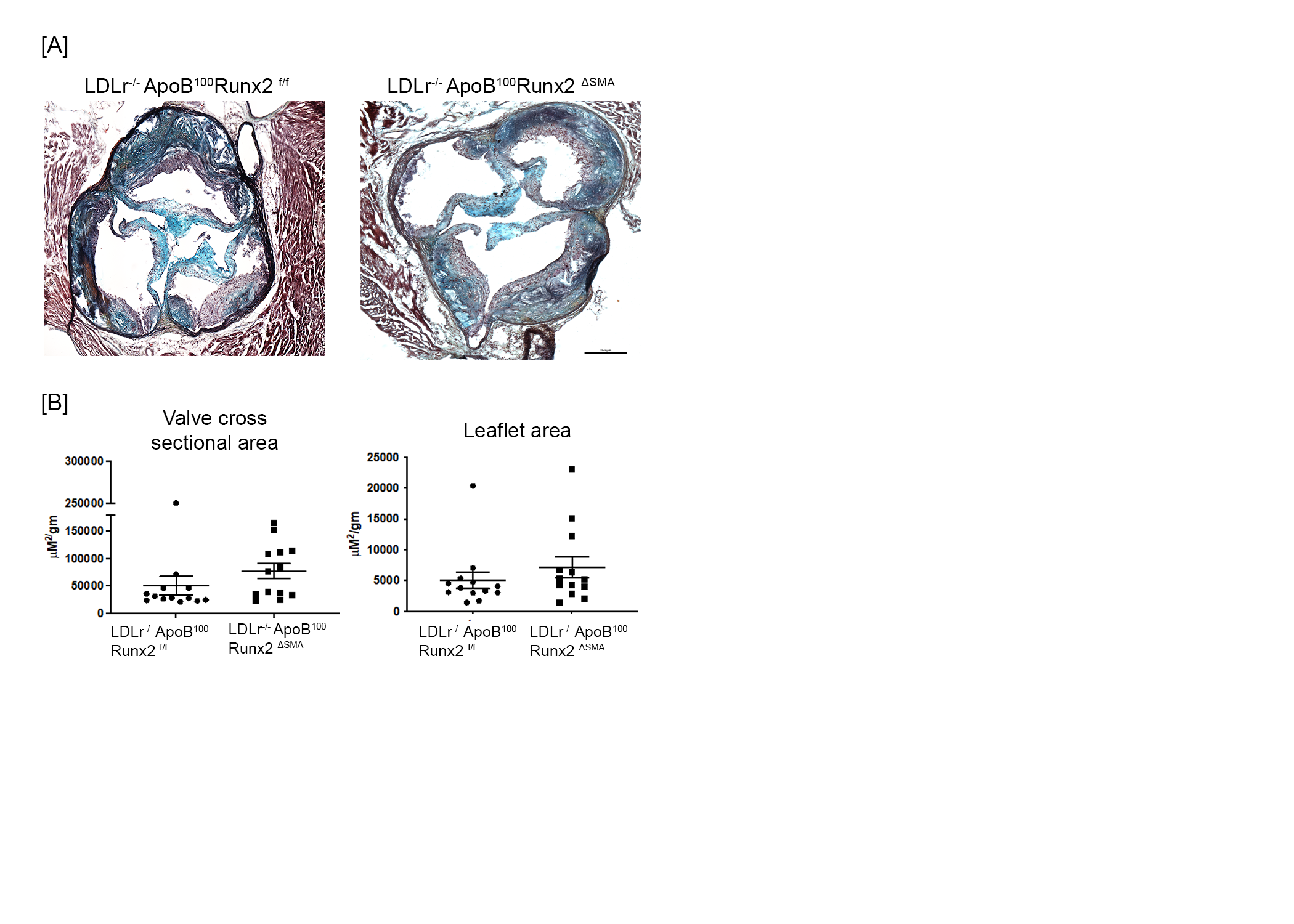

Supplement: Supplementary Figure 3 — Runx2 depletion had no effect on valve cross-sectional area or leaflet area. (A) Representative images of the valve sections stained with Movat pentachrome in LDLr−/−ApoB100Runx2f/f and LDLr−/−ApoB100Runx2ΔSMA mice. (B) Graphs represent the quantification of valve cross-sectional area and leaflet area from Movat stained valve sections normalized to body weight. Differences between groups were analyzed by Mann-Whitney non-parametric tests. Data shown are mean ± SEM. [file Image_3.TIF]
